# Surgery for acute exacerbation of chronic mesenteric ischemia: a case report

**DOI:** 10.1186/s40792-016-0272-0

**Published:** 2016-12-05

**Authors:** Shinji Abe, Tomoji Yamakawa, Hideaki Kawashima, Makoto Yoshida, Setsuji Takanashi, Motoya Kashiyama, Masahiro Ishigooka, Yasushige Shingu, Yoshiro Matsui

**Affiliations:** 1 Department of Cardiovascular Surgery, Kin-i-kyou Central Hospital, 5-1-9-1 Higashinaebo, Higashi-ku, Sapporo, 007-8505 Japan; 2 Department of Surgery, Kin-i-kyou Central Hospital, 5-1-9-1 Higashinaebo, Higashi-ku, Sapporo, 007-8505 Japan; 3 Department of Cardiovascular and Thoracic Surgery, Hokkaido University Hospital, Kita-15, Nishi-7, Kita-ku, Sapporo, 060-8638 Japan

**Keywords:** Chronic mesenteric ischemia, Surgical revascularization

## Abstract

**Background:**

Chronic mesenteric ischemia (CMI) is a rare disease; however, symptomatic CMI has a risk of acute exacerbation without timely revascularization.

**Case presentation:**

A 54-year-old man who had had postprandial pain for 6 months was admitted to our hospital because of vomiting and diarrhea. Although the celiac and superior mesenteric arteries were occluded at the proximal portion, contrast enhancement of the bowel wall was good in contrast-enhanced computed tomography (CECT). Endoscopic examination revealed only a healed gastric ulcer and slight mucosal erosions in the colon. He was diagnosed as having acute enteritis or inflammatory digestive disease and observed with conservative therapy, which improved his acute symptoms. On hospitalization day 42, he suddenly complained of lower back pain. CECT showed abdominal free air, which indicated gastrointestinal perforation. Emergency surgery was performed for jejunum resection. Two days later, a second operation was performed for a leak in the anastomotic site of the jejunum. Necrotic change in the small intestinal serosa was also observed and required broad resection of the small intestine. He was diagnosed with acute exacerbation of CMI, and we performed surgical retrograde bypass to the gastroduodenal artery using a saphenous vein graft as the third operation. After the surgery, he was free from digestive symptoms and was discharged.

**Conclusions:**

When patients complain of chronic and gradual digestive symptoms, we should always consider symptomatic CMI. Timely mesenteric revascularization is important for symptomatic CMI before severe complications occur.

## Background

Chronic mesenteric ischemia (CMI) is an unusual pathology because there is usually abundant collateral circulation among the mesenteric arteries [1–3]. The diagnosis of CMI is sometimes difficult because the symptoms are not specific [4]. Untreated symptomatic CMI can induce severe complications due to exacerbation of intestinal ischemia, which has a poor prognosis [5, 6]. To prevent a poor outcome after acute exacerbation of CMI, timely revascularization is important.

## Case presentation

**Fig. 1:**
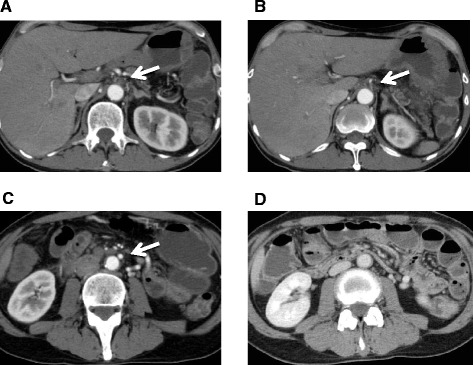
Contrast-enhanced computed tomography findings at the time of admission. The orifices of the celiac (**a**) and superior mesenteric arteries (**b**) are occluded. The inferior mesenteric artery is patent and slightly dilated (**c**). Contrast enhancement of the bowel wall seems normal (**d**)

**Fig. 2:**
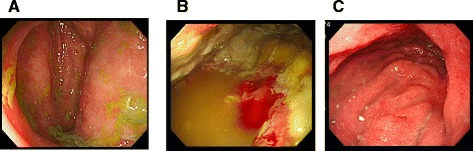
Endoscopic findings. Slight mucosal erosions of the ascending colon at the time of admission (**a**). The gastric mucosa before (**b**) and after (**c**) the revascularization. The necrotic change in the gastric mucosa improved after revascularization

**Fig. 3:**
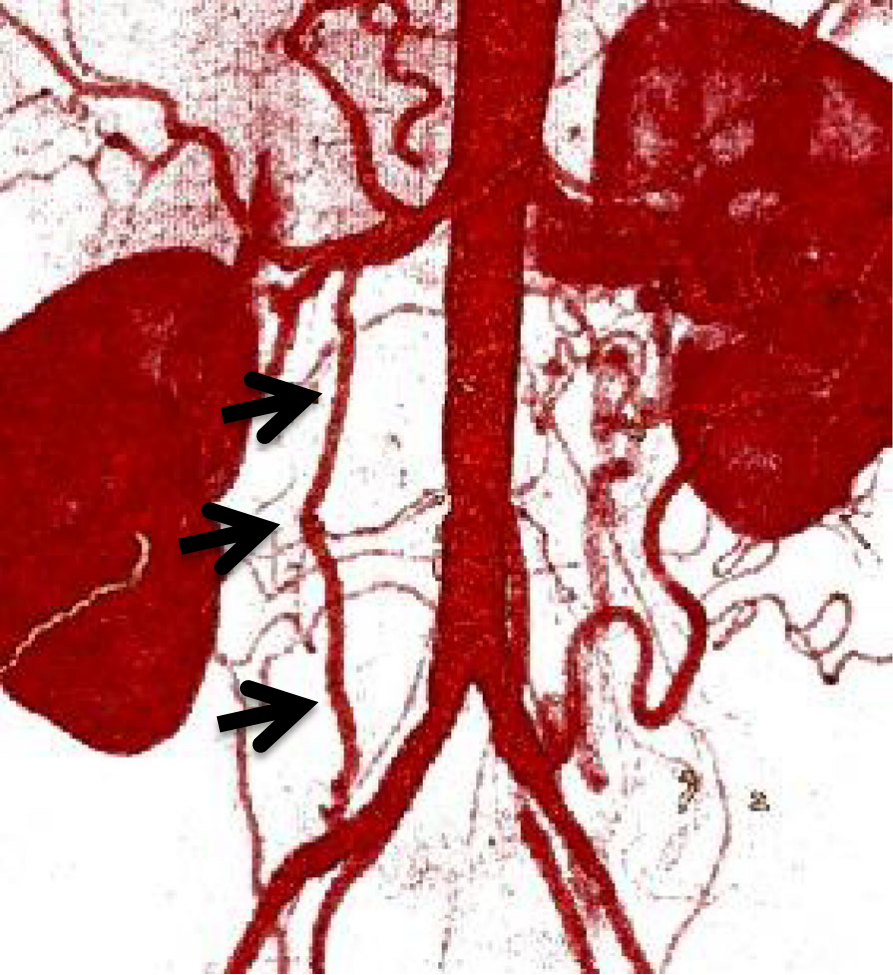
Contrast-enhanced computed tomography finding after retrograde revascularization. The *black arrows* indicate the patent autologous vein graft

A 54-year-old man who had had postprandial pain for 6 months was admitted to our gastroenterology ward because of vomiting and diarrhea over a few days. While he had been performed an abdominal surgery due to intussusception 21 years ago, the detail was not available. He had no other remarkable past history and atherosclerotic risk factors. He complained of tenderness in the lower abdomen without any signs of peritonitis. Blood examination revealed that the levels of white blood cells (13270/μL) and C-reactive protein level (26.7 mg/dL) were elevated. Contrast-enhanced computed tomography (CECT) showed occlusions of both the celiac artery (CA) (Fig. 1a) and the superior mesenteric artery (SMA) (Fig. 1b) in its proximal region. The inferior mesenteric artery (IMA) was patent and slightly dilated (Fig. 1c). Contrast enhancement of the bowel wall was good (Fig. 1d). Endoscopic examination revealed only a healed gastric ulcer and slight mucosal erosions in the cecum, ascending and transverse colon (Fig. 2a). He was treated by conservative therapy under the diagnosis of common acute enteritis during the first 2 weeks; after that, his acute symptoms once improved. However, he presented with recurrence of digestive symptom after starting meals. Endoscopic examination showed progressive ascending and transverse colon ulcers. He was then diagnosed as inflammatory digestive disease and medical therapy was continued. On hospitalization day 42, he suddenly complained of lower back pain. CT showed abdominal free air, which indicated gastrointestinal perforation. Immediately after the diagnosis, emergency surgery was performed. There was no peritonitis in the abdominal cavity. The perforation point was at 60-cm distal to the origin of the jejunum. There was no particular change in the other intestinal serosa. The perforated jejunum was resected and reconstructed. Pathological examination of the resected specimen indicated a possibility of systemic connective tissue disease. However, the definitive diagnosis was not clear. Two days after the emergency surgery, a second operation was performed because of a leak in the anastomotic site of the jejunum. There was also an obvious necrotic change in the small intestinal serosa; therefore, broad resection (120 cm) of the small intestine was required and a jejunostomy was established. Three days after the second procedure, the jejunostomy necrotized. Endoscopic examination detected extended necrosis in the gastroduodenal mucosa (Fig. 2b). Abdominal surgeons diagnosed him as having acute exacerbation of CMI, and they consulted us for mesenteric revascularization for the first time. We first tried an endovascular procedure for the occluded CA and SMA without success. Then we performed retrograde surgical bypass using an autologous saphenous vein and established a new jejunostomy as the third operation. We harvested the saphenous vein graft from his lower extremity. The vein was anastomosed to the right external iliac artery. Another side of the vein was anastomosed to the gastroduodenal artery in an end-to-side fashion. Revascularization of the SMA was not possible because of strong peritoneal adhesion. Blood lactate level was 17 mg/dL after the operation. After the revascularization, we confirmed the graft patency by CECT (Fig. 3) and the recovery of the gastroduodenal mucosa by endoscopic examination (Fig. 2c). His symptoms subsided, and he was discharged from our hospital 62 days after the revascularization. He is now able to eat some meals orally.

## Discussion

CMI is a rare disease because there are usually abundant collateral vessels among the mesenteric arteries [1–3]. Untreated symptomatic CMI can induce acute exacerbation of intestinal ischemia, which has a poor prognosis [5, 6]. Therefore, it is necessary to accurately diagnose and properly treat CMI before severe intestinal ischemia occurs.

### Diagnosis of acute exacerbation of CMI

To diagnose acute exacerbation of CMI correctly, the patient’s symptoms and some imaging modalities are important. The classic symptoms of symptomatic CMI are postprandial pain, food avoidance, and significant weight loss [3]. However, these symptoms are not specific to CMI. It is important to combine analysis of the symptoms and imaging modalities. Imaging modalities include ultrasound (US), endoscopy, CECT, magnetic resonance angiography (MRA), and angiography [7]. In our case, CECT and endoscopy were performed before the mesenteric revascularization. CECT can reveal mesenteric vasculature conditions (calcification, stenosis, and occlusion) and ischemic bowel wall changes (thickening and pneumatosis) [7]. Endoscopy is useful for early detection of intestinal ischemia because intestinal ischemia affects the mucosa first [8]. In our case, the first CECT revealed normal enhancement of the bowel wall, and the endoscopic examination revealed slight erosions of the colon. Because these findings did not indicate severe mesenteric ischemia, it would have been difficult to decide to perform revascularization immediately after admission. However, CECT cannot always reveal definite ischemic changes of the bowel wall in the acute phase [9, 10]. Furthermore, endoscopic findings may not be specific for CMI because other digestive diseases can also induce mucosal changes. These findings of CECT and endoscopy are insufficient to differentiate severe mesenteric ischemia that needs revascularization from other pathologies. To diagnose acute exacerbation of CMI correctly, the combination of analyzing the patient’s symptoms and various imaging modalities is necessary. The prophylactic mesenteric revascularization should have been performed before acute exacerbation to avoid unnecessary broad resection of the intestine because we cannot always predict the acute exacerbation of CMI by standard modalities. In this case, the first intestinal surgical procedure might have expanded the mesenteric ischemia due to injury of the collateral arteries.

### Surgical treatment of mesenteric ischemia

In most cases of chronic mesenteric ischemia, diffuse atherosclerotic disease is associated with its pathology [11]. Therefore, vasodilators, antiplatelet, and anticoagulant drugs may be useful medical management for these patients to avoid symptomatic mesenteric ischemia. However, appropriate surgical intervention would be necessary once patients present with abdominal symptoms. Surgical bypass and endovascular procedures are considered useful treatments for severe mesenteric ischemia [12, 13]. An endovascular procedure was difficult because the CA and SMA were totally occluded at their orifices in our case. In surgical bypass, different roots of grafts are selected—antegrade and retrograde are reported to have equal patency rates [5, 14]. Antegrade revascularization accomplishes less angulated, shorter bypass for CA and SMA, but it is unsuitable for high-risk cases because a supraceliac approach is necessary [15]. Retrograde revascularization is less invasive, but we have to take care about kinking of the grafts due to the longer bypass [15]. Although there are no data indicating that an artificial graft has superior patency to an autologous vein graft, artificial grafts have been reported to be desirable to prevent graft kinking in retrograde revascularization [16]. In our case, we selected retrograde revascularization using an autologous vein graft that had a risk of graft kinking because his abdominal cavity had been contaminated. We currently recommend prophylactic mesenteric revascularization using an artificial graft in the case of CMI based on this experience. Single- versus multiple-vessel revascularizations is another controversial issue. While Hollier et al. reported that complete revascularization reduced symptomatic recurrences [17], Park et al. reported that the recurrence rate of symptoms was not different between single- and multiple-vessel revascularizations [4]. In our case, strong peritoneal adhesion made the revascularization to the SMA difficult. Anastomosis only to the gastroduodenal artery, a branch of the CA, improved his symptoms. We presume that the network between the CA and the SMA played an important role for collateral blood supply to the SMA.

## Conclusions

It is difficult to diagnose acute exacerbation of CMI by CT and endoscopy only. When patients with a stenotic CA or SMA complain of chronic and gradual abdominal symptoms, we should always consider symptomatic CMI, which needs timely mesenteric revascularization before severe intestinal ischemia occurs.

## Abbreviations

CA: Celiac artery
CECT: Contrast-enhanced computed tomography
CMI: Chronic mesenteric ischemia
IMA: Inferior mesenteric artery
SMA: Superior mesenteric artery

## References

[1] Zeller T, Rastan A, Sixt S (2010). Chronic atherosclerotic mesenteric ischemia (CMI). Vasc Med.

[2] McAfee MK, Cherry KJ, Naessens JM, Pairolero PC, Hallett JW, Gloviczki P (1992). Influence of complete revascularization on chronic mesenteric ischemia. Am J Surg.

[3] Chandra A, Quinones-Baldrich WJ (2010). Chronic mesenteric ischemia: how to select patients for invasive treatment. Semin Vasc Surg.

[4] Park WM, Cherry KJ, Chua HK, Clark RC, Jenkins G, Harmsen WS (2002). Current results of open revascularization for chronic mesenteric ischemia: a standard for comparison. J Vasc Surg.

[5] Park WM, Gloviczki P, Cherry KJ, Hallett JW, Bower TC, Panneton JM (2002). Contemporary management of acute mesenteric ischemia: factors associated with survival. J Vasc Surg.

[6] Yasuhara H (2005). Acute mesenteric ischemia: the challenge of gastroenterology. Surg Today.

[7] Hohenwalter EJ (2009). Chronic mesenteric ischemia: diagnosis and treatment. Semin Interv Radiol.

[8] Acosta S (2014). Surgical management of peritonitis secondary to acute superior mesenteric artery occlusion. World J Gastroenterol.

[9] Lehtimäki TT, Kärkkäinen JM, Saari P, Manninen H, Paajanen H, Vanninen R (2015). Detecting acute mesenteric ischemia in CT of the acute abdomen is dependent on clinical suspicion: review of 95 consecutive patients. Eur J Radiol.

[10] Ouchi A, Isogai M, Harada T, Kaneoka Y, Kamei K, Maeda A (2014). Duodenal ulcer penetration into the superior mesenteric artery after percutaneous transluminal angioplasty and stent placement for acute mesenteric ischemia: report of a case. Surg Today.

[11] Mastoraki A, Mastoraki S, Tziava E, Touloumi S, Krinos N, Danias N (2016). Mesenteric ischemia: pathogenesis and challenging diagnostic and therapeutic modalities. World J Gastrointest Pathophysiol.

[12] Acosta S, Björck M (2014). Modern treatment of acute mesenteric ischaemia. Br J Surg.

[13] Malgor RD, Oderich GS, McKusick MA, Misra S, Kalra M, Duncan AA (2010). Results of single and two-vessel mesenteric artery stents for chronic mesenteric ischemia. Ann Vasc Surg.

[14] Doyle AJ, Chandra A (2012). Chronic mesenteric ischemia in a 26-year-old man: multivessel median arcuate ligament compression syndrome. Ann Vasc Surg.

[15] Oderich GS, Gloviczki P, Bower TC (2010). Open surgical treatment for chronic mesenteric ischemia in the endovascular era: when it is necessary and what is the preferred technique?. Semin Vasc Surg.

[16] Foley MI, Moneta GL, Abou-Zamzam AM, Edwards JM, Taylor LM, Yeager RA (2000). Revacularization of the superior mesenteric artery alone for treatment of intestinal ischemia. J Vasc Surg.

[17] Hollier LH, Bernatz PE, Pairolero PC, Payne WS, Osmundson PJ (1981). Surgical management of chronic intestinal ischemia: a reappraisal. Surgery.

